# Loss of *Frrs1l* disrupts synaptic AMPA receptor function, and results in neurodevelopmental, motor, cognitive and electrographical abnormalities

**DOI:** 10.1242/dmm.036806

**Published:** 2019-02-22

**Authors:** Michelle Stewart, Petrina Lau, Gareth Banks, Rasneer Sonia Bains, Enrico Castroflorio, Peter L. Oliver, Christine L. Dixon, Michael C. Kruer, Dimitri M. Kullmann, Abraham Acevedo-Arozena, Sara E. Wells, Silvia Corrochano, Patrick M. Nolan

**Affiliations:** 1 MRC Harwell Institute, Harwell Campus, Oxfordshire OX11 0RD, UK; 2 Department of Clinical and Experimental Epilepsy, UCL Institute of Neurology, Queen Square, London WC1N 3BG, UK; 3 Barrow Neurological Institute, Phoenix Children's Hospital, Phoenix, AZ 85013, USA; 4 Unidad de Investigación Hospital Universitario de Canarias, La Laguna 38320, Spain; 5 ITB, Universidad de La Laguna, La Laguna 38320, Spain; 6 Network Center for Biomedical Research in Neurodegenerative Diseases (CIBERNED), La Laguna 38320, Spain

**Keywords:** AMPA receptors, Behaviour, *Frrs1l*, Mouse model, Seizures

## Abstract

Loss-of-function mutations in a human AMPA receptor-associated protein, ferric chelate reductase 1-like (FRRS1L), are associated with a devastating neurological condition incorporating choreoathetosis, cognitive deficits and epileptic encephalopathies. Furthermore, evidence from overexpression and *ex vivo* studies has implicated FRRS1L in AMPA receptor biogenesis, suggesting that changes in glutamatergic signalling might underlie the disorder. Here, we investigated the neurological and neurobehavioural correlates of the disorder using a mouse *Frrs1l* null mutant. The study revealed several neurological defects that mirrored those seen in human patients. We established that mice lacking *Frrs1l* suffered from a broad spectrum of early-onset motor deficits with no progressive, age-related deterioration. Moreover, *Frrs1l^−/−^* mice were hyperactive, irrespective of test environment, exhibited working memory deficits and displayed significant sleep fragmentation. Longitudinal electroencephalographic (EEG) recordings also revealed abnormal EEG results in *Frrs1l^−/−^* mice. Parallel investigations into disease aetiology identified a specific deficiency in AMPA receptor levels in the brain of *Frrs1l^−/−^* mice, while the general levels of several other synaptic components remained unchanged, with no obvious alterations in the number of synapses. Furthermore, we established that *Frrsl1* deletion results in an increased proportion of immature AMPA receptors, indicated by incomplete glycosylation of GLUA2 (also known as GRIA2) and GLUA4 (also known as GRIA4) AMPA receptor proteins. This incomplete maturation leads to cytoplasmic retention and a reduction of those specific AMPA receptor levels in the postsynaptic membrane. Overall, this study determines, for the first time *in vivo*, how loss of FRRS1L function can affect glutamatergic signalling, and provides mechanistic insight into the development and progression of a human hyperkinetic disorder.

This article has an associated First Person interview with the first author of the paper.

## Abstract (precis)

**Summary:** Loss of the epilepsy-related gene *Frrs1l* in mice causes a dramatic reduction in AMPA receptor levels at the synapse, eliciting severe motor and coordination disabilities, hyperactivity and cognitive defects, with some evidence of behavioural seizures.

## INTRODUCTION

Ferric chelate reductase 1-like (FRRS1L) is a novel, highly conserved, brain-specific protein, the functional characterisation of which has only recently been under investigation. Studies in patients have found nine families with recessive mutations in *FRRS1L*, which result in severe intellectual disability, movement disorders, hypotonia and epilepsy (Madeo et al., 2016; Shaheen et al., 2016; Brechet et al., 2017). In some patients, these clinical symptoms are accompanied by neurodegeneration in the cortex and cerebellum. Several families have now been diagnosed with this devastating condition, arguing for the inclusion of this gene in the diagnostic screening for epilepsy and dyskinetic disorders (Carecchio and Mencacci, 2017). The dramatic clinical consequences of carrying mutations in this gene point to an important neurological function for *FRRS1L*, which has not yet been elucidated, hence challenging efforts in therapeutic development.

Although named for its sequence similarity to ferric chelate reductase 1, FRRS1L has only a poorly characterised dopamine beta-monooxygenase N-terminal (DOMON) domain (IPR005018) and a transmembrane domain, with the ferric chelate reductase domain being absent; therefore, its function is likely to be distinct from that of its namesake, FRRS1. *Frrs1l* is expressed in the central nervous system (CNS) and testes of adult mice and in developing embryonic forebrain (Madeo et al., 2016). Further expression analysis in the adult mouse brain shows *Frrs1l* expression in the excitatory neurons in the cerebral cortex, hippocampus and midbrain, medium spiny neurons in the striatum, granule cells in the dentate gyrus and Purkinje cells in the cerebellum (Zeisel et al., 2018). Emerging studies have begun to unravel the role of FRRS1L in the CNS, importantly, in α-amino-3-hydroxy-5-methyl-4-isoxazolepropionic acid (AMPA) receptor complex function. FRRS1L colocalises with calnexin in the endoplasmic reticulum (ER) of rat hippocampal neurons (Brechet et al., 2017). Results of knockdown and exogenous overexpression studies in cultured hippocampal neurons suggest that FRRS1L, along with carnitine palmytoyltransferase 1c (CPT1C), is involved in the early stages of AMPA receptor complex biogenesis, binding to the core AMPA proteins, GLUA1-4 (also known as GRIA1-4), but dissociating before the final auxiliary proteins bind to make a functional receptor (Brechet et al., 2017). Furthermore, reduction of FRRS1L levels in cultured hippocampal neurons leads to an overall decrease in AMPA receptor levels, as well as to modifications in synaptic transmission. In addition, interactions with dynein complex proteins suggest a potential role for FRRS1L in dynein-based AMPA trafficking (Han et al., 2017).

AMPA receptors are essential ionotropic glutamate receptors and mediate much of the fast-excitatory synaptic transmission in the brain. AMPA receptors are composed of four core proteins, GLUA1-4, which form a heterotetrameric complex at the centre of the receptor. Associated with this core complex are a variety of auxiliary subunits with distinct roles in the maturation of AMPA receptors (Chen et al., 2000; Tomita et al., 2003; Kato et al., 2010; Schwenk et al., 2012, 2014; Erlenhardt et al., 2016). These auxiliary proteins have distinct roles in regulating the spatiotemporal activity of AMPA receptors; however, many of these roles have yet to be elucidated.

The majority of human variants in patients with homozygous mutations in *FRRS1L* are predicted to lead to a premature stop codon and loss of the transmembrane domain, consequently leading to a loss of function. A knockout of the murine *Frrs1l* gene (*Frrs1l^tm1b/tm1b^*) has been generated by the International Mouse Phenotyping Consortium (IMPC, http://www.mousephenotype.org) to investigate the consequences of *Frrs1l* loss *in vivo*. Initial characterisation of this line uncovered a range of aberrant phenotypes including hyperactivity, abnormal gait, decreased grip strength and partial pre-weaning lethality (Koscielny et al., 2014; http://www.mousephenotype.org, accessed 01-06-2018). In the current study, we use this mouse line to explore its validity as a model and to investigate the phenotypic deficits in more depth. Moreover, we make use of this model to study whether a disturbance in AMPA receptor maturation is the mechanism underlying the pathology of the disorder, providing new *in vivo* evidence for the pivotal role that *Frrs1l* has in AMPA receptor physiology.

## RESULTS

### Loss of *Frrs1l* results in increased neonatal lethality, smaller size and early-onset motor deficits

**Fig. 1.**
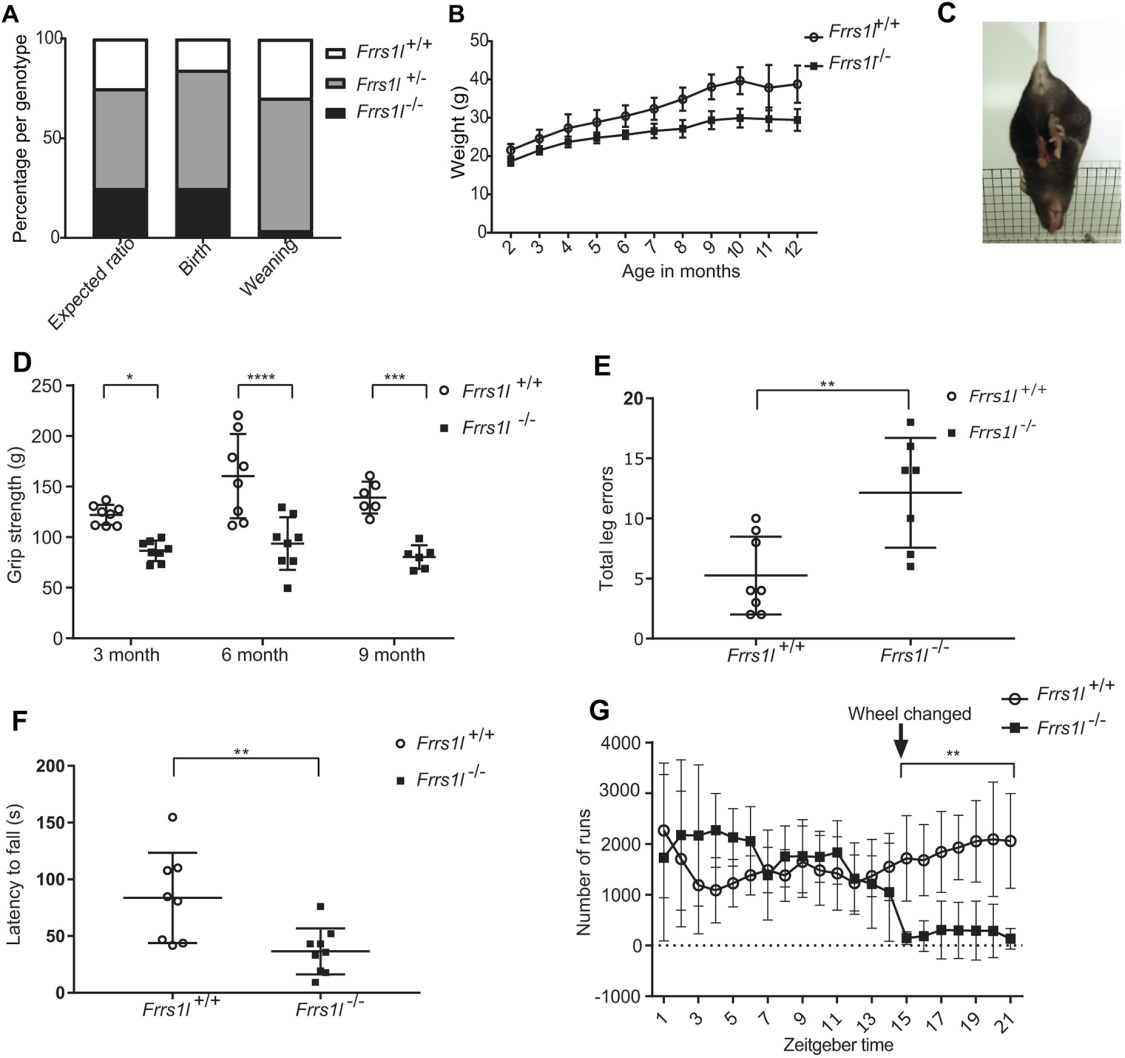
***Frrs1l^−/−^* have decreased survival and body weight, and coordination and limb-grasping abnormalities.** (A) *Frrs1l^−/−^* are born in accordance with predicted Mendelian ratios (*P*=0.43) and show no significant difference from expected numbers. Pups were genotyped at weaning and greatly reduced numbers of *Frrs1l^−/−^* were found (*P*<0.0001). Data analysed by chi-squared test. (B) *Frrs1l^−/−^* have significantly lower weight than controls from 6 months of age but show a similar weight curve and no deterioration. Data analysed by two-way ANOVA with post hoc comparisons. *n*=7 *Frrs1l^+/+^* at 2-11 months; *n*=5 *Frrs1l^+/+^* at 12 months; *n*=9 *Frrs1l^−/−^* at 2-4 months; *n*=8 *Frrs1l^−/−^* at 5 months; *n*=7 *Frrs1l^−/−^* at 6-8 months; *n*=6 *Frrs1l^−/−^* at 9-12 months. (C) *Frrs1l^−/−^* show increased incidence of limb grasping (*P*<0.01 at 3, 6 and 9 months); a representative image is shown. (D) Grip strength of *Frrs1l^−/−^* is significantly reduced at 3, 6 and 9 months compared with that of wild-type littermate controls. Data analysed by repeated measures ANOVA followed by Sidak multiple comparisons test; data are the average of three trials at each time point. *n*=8 *Frrs1l^+/^*, *n*=8 *Frrs1l^−/−^* at 3 and 6 months; *n*=6 *Frrs1l^+/+^*, *n*=6 *Frrs1l^−/−^* at 9 months. (E) Misplacement of feet on the Locotronic horizontal ladder results in an increased number of errors in *Frrs1l^−/−^*. *n*=8 *Frrs1l^+/+^*, *n*=7 *Frrs1l^−/−^*. Data analysed using general linear model with Poisson distribution. (F) *Frrs1l^−/−^* have a significantly decreased latency to fall from an accelerating rotarod (*P*<0.01) (*n*=8 *Frrs1l^+/+^*, *n*=8 *Frrs1l^−/−^*). Data analysed by repeated measures ANOVA. (G) *Frrs1l^−/−^* display no difference in wheel running on simple wheels but show a significant difference when changed to complex wheels, analysed using ANOVA followed by Sidak multiple comparisons test. *n*=5 *Frrs1l^+/+^*, *n*=5 *Frrs1l^−/−^*. *P<0.05, **P<0.01, ***P<0.001, ****P<0.0001. Body weight (B) and wheel-running (G) data are mean±s.d. All error bars indicate s.d.

*Frrs1l^−/−^* mice are born at expected Mendelian ratios; milk is present in the stomach, breathing is apparently normal, and it is not possible to visibly distinguish between *Frrs1l^−/−^* and wild-type littermates. However, >90% of *Frrs1l^−/−^* neonates die between 12 h and 24 h after birth. Analysis of numbers per genotype at weaning shows a difference in expected ratios (*P*<0.0001), whereas at postnatal day (P) 0 the ratio of genotypes is not significantly different from that expected (*P*=0.43) (Fig. 1A). Tissue was collected from any pups that were found dead in the first days after birth and genotyping carried out. We found mortality to be higher in *Frrs1l^−/−^*, with a greater proportion of homozygotes being found dead than would be expected by chance if this was not a genotype effect (*P*<0.001). Gross pathology was performed on pups at P0 and no obvious abnormalities were found in 44 tissues examined (data not shown). *Frrs1l^−/−^* mice that survived past P2 continued to thrive to weaning and beyond. Five of the nine female homozygous mice were killed during the course of the study as they reached previously specified humane endpoints, including seizure without full recovery (*n*=1), self-inflicted wounds and stereotypical behaviour (*n*=2), uncoordinated gait impinging on the ability to feed (*n*=1) and breathing difficulties (*n*=1).

In order to confirm that homozygous mutant mice no longer express *Frrs1l*, we conducted quantitative PCR (qPCR) assays with primers spanning all five exons of the gene, including the targeted exon 3. We confirmed no significant expression of *Frrs1l* in P0 and adult *Frrs1l^−/−^* brains compared with wild-type littermates (Fig. S1A,B). Of the *Frrs1l^−l−^* mice that survived to weaning, all showed prominent reduced body weight when compared with littermate controls (*P*<0.05 from 6 months onwards) (Fig. 1B). However, the weight curve is not dissimilar to that of wild type and does not show significant decline with age up to 14 months, suggesting a neurodevelopmental effect rather than a progressive wasting phenotype. Given the difference in body size, we further examined archived IMPC x-ray images and found that *Frrs1^−l−^* mice have a significantly shorter tibia length, and therefore smaller body size, than wild-type controls for both females (*P*<0.01) and males (*P*<0.05) (http://www.mousephenotype.org, 2015). IMPC data also show no differences in body composition or calorimetric measurements of metabolic rate. In some cases, several years after the onset of symptoms, human patients carrying mutations in *FRRS1L* express cerebellar atrophy and other pathological alterations in the brain (Madeo et al., 2016). In *Frrs1l^−/−^* cohorts, we found total brain weight to be ∼10% less than that of wild-type controls (*Frrs1l^+/+^*, 0.459±0.018 g; *Frrs1l^−/−^*, 0.417±0.009 g) (*P*<0.05); however, no gross anatomical pathologies were evident. Moreover, brain size differences were not significant after normalisation for body weight.

Animals that survived through early postnatal development were assessed using a focused battery of physiological, behavioural and motor function tests throughout adulthood.

Motor phenotyping was carried out to determine whether *Frrs1l^−/−^* mice displayed abnormalities in movement and muscle force similar to those described in humans carrying *FRRS1L* mutations. We used a standard battery of tests, including SmithKline Beecham, Harwell Imperial College, Royal London Hospital phenotype assessment (SHIRPA), grip strength and rotarod, followed by more complex testing of motor function using a horizontal ladder and a 3-week trial that measures progressive wheel-running performance parameters. On visual inspection, *Frrs1l^−/−^* mice have an abnormal gait, which is evident at weaning, and, in SHIRPA, show additional significant differences from wild-type littermate controls when assessed at 3, 6 and 9 months of age (*P*<0.05, *P*<0.01 and *P*<0.001, respectively) (Table 1).

**Table 1.**
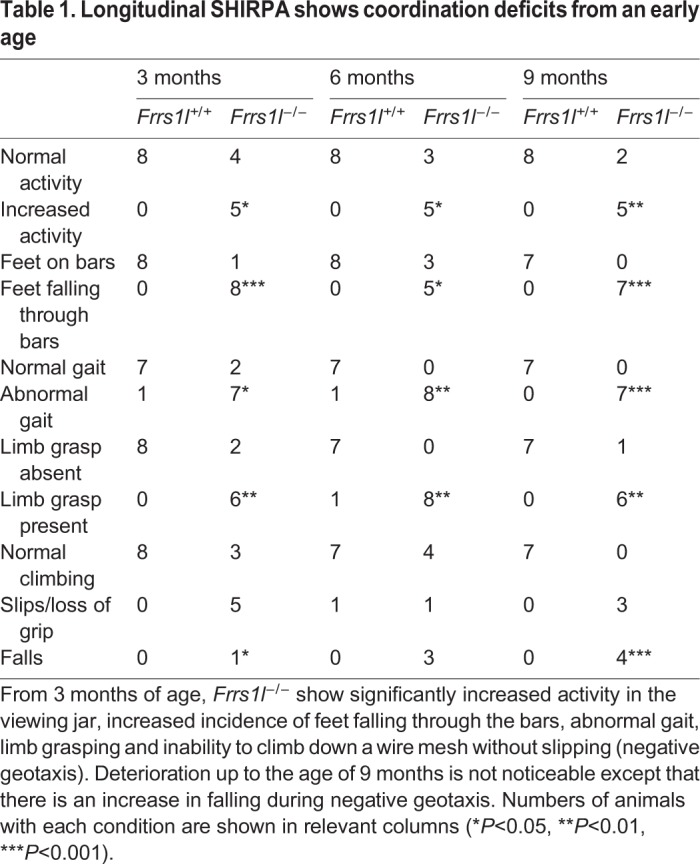
**Longitudinal SHIRPA shows coordination deficits from an early age**

Abnormalities highlighted in SHIRPA include lack of coordination, demonstrated by inability to place feet correctly on a grid floor (*P*<0.001 at 9 months), loss of grip when climbing down a vertical grid (*P*<0.001 at 9 months) and reduced muscle force associated with an increased incidence of limb grasping (*P*<0.01 at all time points) (Table 1, Fig. 1C). Limb grasping is also associated with defects in many neurological disorders, and it may indicate alterations in corticostriatal circuits (Lalonde and Strazielle, 2011).

We measured grip strength at 3, 6 and 9 months of age to confirm quantitatively that the loss of *Frrs1l* resulted in reduced ability to grip. Indeed, grip force in all four limbs was significantly reduced in *Frrs1l^−/−^* mice compared with wild-type controls (Fig. 1D). The effect remained significant even after correcting for weight differences (3 months, *P*<0.05; 6 months, *P*<0.05; 9 months, *P*<0.01). This loss of ability to grip is expressed from an early age without further deterioration, suggesting a developmental non-progressive phenotype.

Next, we assessed gait and motor coordination by performing three complementary tests at a single time point for each: a horizontal ladder challenge (Locotronic), a rotarod test and a wheel-running paradigm. In the Locotronic challenge, *Frrs1l^−/−^* mice showed an increased frequency of errors (misplacement of feet) whilst moving along the horizontal ladder compared with their littermate controls (*P*<0.01) (Fig. 1E). This elaborates upon previous observations in SHIRPA, in which homozygotes had a higher number of instances of feet falling through the bars of the grid.

Supporting these data, *Frrs1l^−/−^* mice also had a shorter latency to fall when placed on an accelerating rotarod (*P*<0.05) (Fig. 1F). In the motor function assessment by wheel running, no differences were observed between *Frrs1l^−/−^* and wild-type mice during the first 2 weeks. In the third week, the standard wheel was removed and replaced by a complex wheel with rungs missing at uneven intervals. After the new challenge was introduced, *Frrs1l^−/−^* were unable to run at all, showing a drop in running attempts to almost zero for the remainder of the third week (*P*<0.001) (Fig. 1G).

Thus, all tests indicate that mice lacking the *Frrs1l* gene suffer from a dramatic loss in grip strength with loss of motor coordination and motor disabilities from an early age.

### *Frrs1l^−/−^* mice are hyperactive, and have cognitive deficits and abnormalities in immobility-defined sleep

**Fig. 2.**
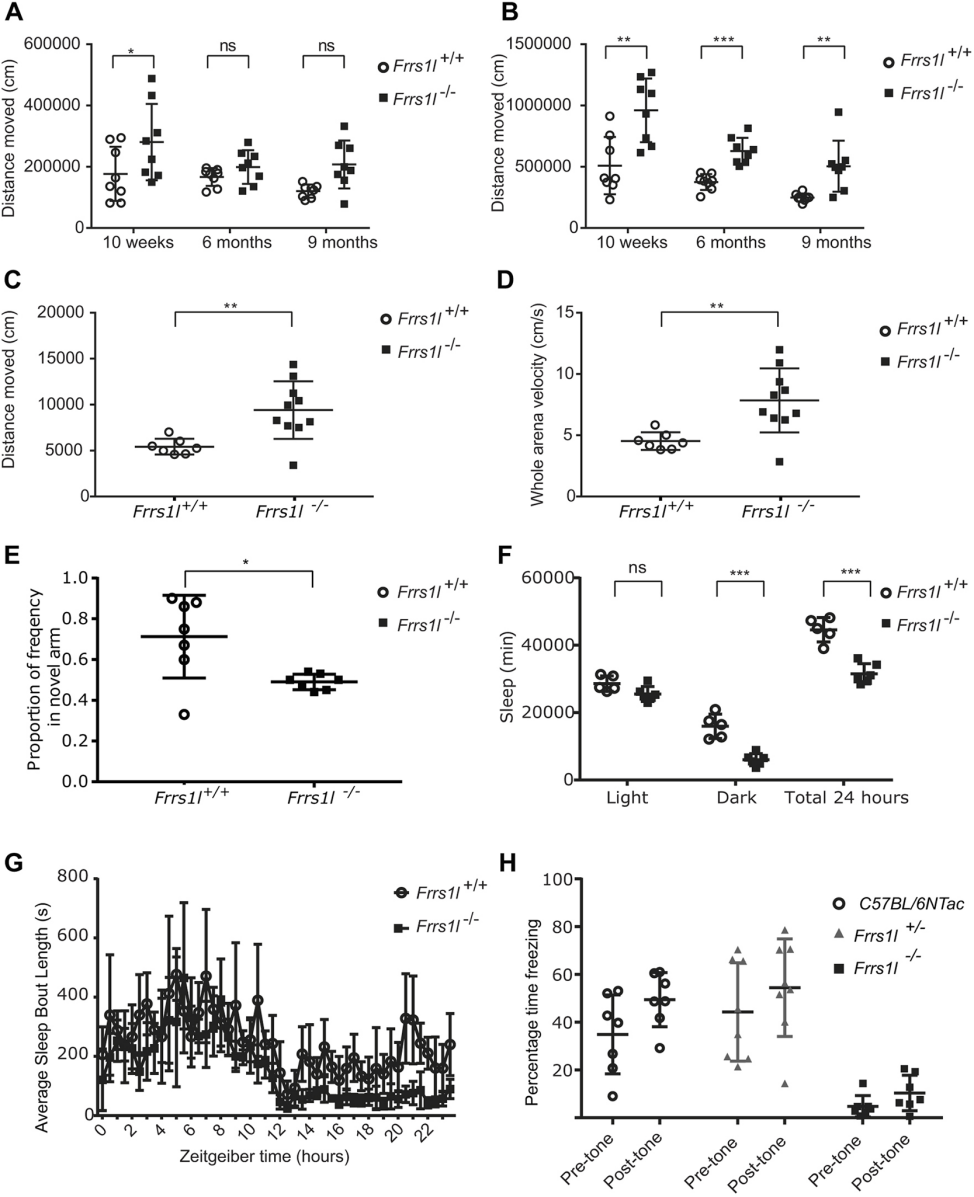
**Deletion of *Frrs1l* causes hyperactivity, working memory deficits and abnormal sleep pattern.** (A,B) An increase in distance moved is apparent in the home cage both during the light phase (A) and the dark phase (B) at 10 weeks old (*P<0.05, **P<0.01), and only in the dark phase at 6 months (***P<0.001) and 9 months (**P<0.01). Data analysed by ANOVA followed by Sidak multiple comparisons test. *n*=8 *Frrs1l^+/+^*, *n*=8 *Frrs1l^−/−^*. (C,D) Total distance moved (C) and velocity in an open-field arena (D) are significantly increased in *Frrs1l^−/−^* compared with *Frrs1l^+/+^* controls (**P<0.01). Data analysed by Student's *t*-test. *n*=8 *Frrs1l^+/+^*, *n*=8 *Frrs1l^−/−^*. (E) *Frrs1l^−/−^* do not show a preference for the novel arm in a forced alternation Y-maze task. Frequency of *Frrs1l^−/−^* entry into the novel arm stays at chance level, while *Frrs1l^+/+^* show increased exploration. Data are ratio of frequency in novel arm [frequency in novel arm/(frequency in novel arm–frequency in familiar arm)]. *n*=7 *Frrs1l^+/+^*, *n*=7 *Frrs1l^−/−^* (*P<0.05). Data analysed using Student's *t*-test. (F) *Frrs1l^−/−^* exhibit abnormal immobility-defined sleep behaviour. *Frrs1l^−/−^* show decreased total time asleep in a 24-h period, with the total time asleep in the dark phase being significantly reduced (***P<0.001). (G) Sleep bout length is significantly reduced in *Frrs1l^−/−^*. *Frrs1l^−/−^* have a fragmented sleep pattern, with less time asleep overall and shorter sleep bouts. *n*=5 *Frrs1l^+/+^*, *n*=6 *Frrs1l^−/−^*. Data are mean±s.d. (H) A separate cohort of *Frrs1l^−/−^* males, assessed as part of the IMPC pipeline, do not show an increase in freezing in response to tone during a cued fear conditioning paradigm. The difference between pre-cue and post-cue freezing was significantly lower than in controls (*P*=0.004). ns, non-significant. All error bars indicate s.d.

Previous IMPC-based assessment of these mice had indicated a hyperactivity phenotype. To confirm and further define this hyperactivity, mice were assessed at a number of time points in group-housed conditions in the home cage to evaluate their activity continuously in an undisturbed, non-stressful environment. *Frrs1l^−/−^* mice displayed increased activity when recorded at 10 weeks of age throughout both light and dark phases (*P*<0.05 light, *P*<0.0001 dark) and increased activity at 6 and 9 months in the dark phase only (*P*<0.05 at both time points) (Fig. 2A,B). These home-cage data indicate that the hyperactivity is not a consequence of being introduced to a novel environment.

In the novel environment of the open-field test at a single time point, *Frrs1l^−/−^* mice exhibited increased activity in the whole arena, demonstrated by a greater total distance moved (*P*<0.01) (Fig. 2C) and an increased velocity (*P*<0.01) (Fig. 2D), supporting the hyperactivity phenotype observed in the home-cage analysis. The frequency to enter the centre of the arena and velocity in the centre of the arena were also significantly increased (*P*<0.05); however, distance moved and duration in the centre were not different between *Frrs1l^−/−^* and wild type, indicating that the mice are hyperactive, although we cannot rule out the contribution of an altered anxiety state in mutants (Fig. S2A).

Learning disabilities are among the common features associated with intellectual disability, such as those seen in patients carrying mutations in *FRRS1L*. In the null mice, we evaluated working memory using the Y-maze forced alternation test. Interestingly, *Frrs1l^−/−^* mice showed no preference for the novel arm (Fig. 2E) (*P*<0.05), suggesting a working memory deficit.

Previous studies have demonstrated that defects in AMPA receptor composition are associated with both intellectual disability and perturbed sleep patterns (Davies et al., 2017). Since FRSS1L has been proposed to play a role in AMPA receptor assembly, we assessed sleep status in *Frrs1l^−/−^* animals using passive infrared movement tracking (PIR) (Brown et al., 2016), which scores sleep using periods of immobility as a behavioural sleep correlate. The total immobility-defined sleep of *Frrs1l^−/−^* animals was significantly less than that of wild-type controls in the dark phase of the light/dark cycle (time spent asleep in the dark, *P*=0.0002) (Fig. 2F). Additionally, the average length of sleep bouts was significantly reduced in *Frrs1l^−/−^* animals (average sleep bout length, *P*=0.001). Analysis of sleep bout length in the light and dark phases of the light/dark cycle revealed that *Frrs1l^−/−^* animals had a significant reduction in sleep bout length in the dark phase of the light cycle, with no significant effect in the light phase (average sleep bout length in the dark, *P*=0.00001; average sleep bout length in the light, *P*=0.079) (Fig. 2G). Interestingly, *Frrs1l^−/−^* mice show no overt changes in circadian period or entrainment (data not shown).

We also examined the IMPC archive data on the fear conditioning paradigm, a test used to measure cognitive abilities, specifically those associated with non-declarative memory formation (LaBar and Cabeza, 2006). Mice lacking FRRS1L have deficits in cued, but not contextual, fear conditioning, demonstrating an inability to learn the association between a tone and an aversive stimulus and therefore an impairment in implicit memory (Fig. 2H).

### Loss of *Frrs1l* causes abnormal EEG

Mutations in *FRRS1L* are associated with epileptic encephalopathy*.* Behavioural seizures were observed during phenotyping on several occasions, consisting of episodes of behavioural arrest and lordotic posture in *Frrs1l^−/−^* animals, as well as one mouse dying during a generalised convulsion. We carried out electroencephalographic (EEG) recordings in *Frrs1l^−/−^* animals to ascertain whether the episodes seen could represent seizures. After the implantation of EEG transmitters in adult mice (*n*=2 *Frrs1l^/+/+^*, *n*=3 *Frrs1l^−/−^*, *n*=2 C57BL/6NTac), we recorded EEG activity in their home-cage environment for 5-15 days.

**Fig. 3.**
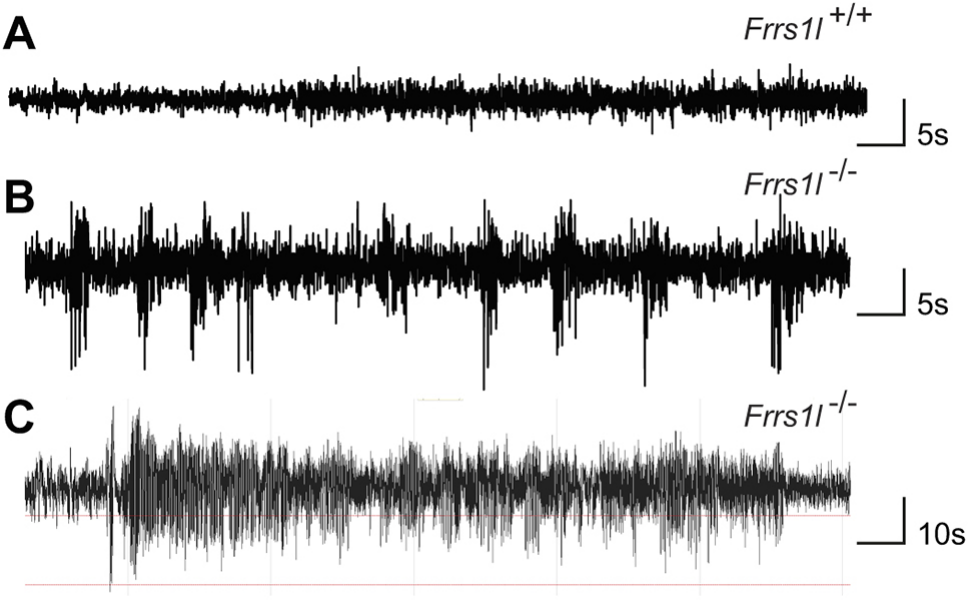
**EEG abnormalities are seen in *Frrs1l^−/−^* animals.** (A) EEG sample extracted from a FRRS1L wild-type littermate control. (B,C) Representative traces from different *Frrs1^−/−^* animals showing repeated runs of polyspikes (B) and a seizure-like episode (C).

Although we did not capture discrete electrographic seizures, *Frrs1l^−/−^* mice exhibited clear evidence of encephalographic abnormalities that were not observed in wild-type controls (C57BL/6NTac and *Frrs1l^/+/+^* animals) (Fig. 3). We are aware of the limitations of this assessment and quantifiable analysis of EEG warrants further investigation. These results are consistent with the view that loss of *Frrsl1* leads to a profound encephalopathy.

### Decreased AMPA receptor protein levels in *Frrs1l^−/−^* brain

**Fig. 4.**
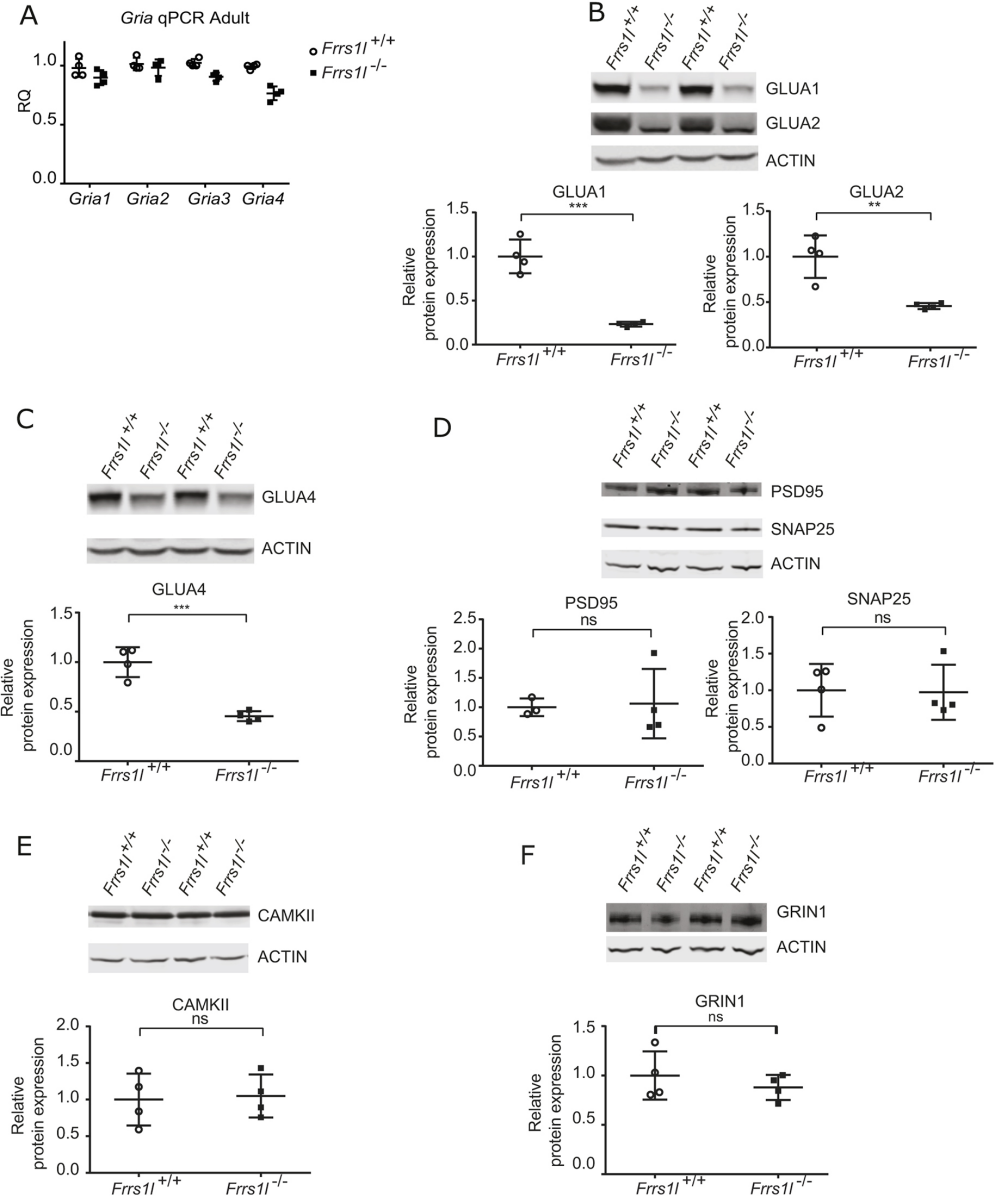
***Frrs1l* deficiency leads to changes in AMPA receptor subunit levels with no change in gene expression.** (A) qPCR for *Gria1-4* shows no changes between *Frrs1l^−/−^* and wild-type controls for *Gria1* and *Gria2*, with only slight but significant changes for *Gria3* and *Gria4* (*P*<0.05 and *P*<0.001, respectively). (B,C) GLUA1, GLUA2 (B) and GLUA4 (C) immunoreactivities are all significantly lower in *Frrs1l^−/−^* adult cerebellum than in control adult cerebellum (**P<0.01, ***P<0.001). The patterns of GLUA2 and GLUA4 immunoreactivity differ between *Frrs1l^−/−^* and wild-type controls, with a diffuse band present for GLUA2 in wild type, but only a thin band in *Frrs1l^−/−^*. GLUA4 blots also indicate changes in immunoreactive band mobility. (D-F) Other synaptic proteins – PSD95, SNAP25 (D) and CAMKII (E) and NMDAR (GRIN1) (F) – remain unchanged between *Frrs1l^−/−^* and wild-type controls. Data analysed using Student's *t*-test (*n*=4 *Frrs1l^+/+^*, *n*=4 *Frrs1l^−/−^*). ns, non-significant.

In order to understand the mechanisms underlying the neurological and behavioural deficits described above, we investigated whether AMPA receptor levels were altered *in vivo* as a consequence of *Frrs1l* deletion, as suggested in earlier *in vitro* studies (Brechet et al., 2017). We first examined whether the deletion of *Frss1l* would cause alterations in the gene expression levels of the four core AMPA receptor genes (*Gria1-4*) and found no differences between wild-type and *Frrs1l^−/−^* mice in P0 brain or in adult brain (Fig. 4A; Fig. S1C). We next examined levels of three core AMPA receptor proteins (GLUA1, GLUA2 and GLUA4) in P0 brain and in 14-month-old cerebellum. In adult cerebellum, GLUA1, GLUA2 and GLUA4 levels were all significantly reduced in *Frrs1l^−/−^* compared with wild-type controls (*P*<0.001, *P*<0.01 and *P*<0.001, respectively) (Fig. 4B,C). In P0 brain, only GLUA1 was significantly reduced, while alterations in immunoreactive band mobility were noted for both GLUA2 and GLUA4 (Fig. S3B).

To confirm that there was a specific loss of AMPA receptors in homozygotes rather than a general loss in synaptic number, we compared the levels of several standard synaptic proteins in adult cerebellum and P0 brain. Interestingly, we found no differences in expression of any of the synaptic proteins assessed [CAMKII, SNAP25, PSD95 (also known as DLG4) and *N*-methyl-D-aspartate (NMDA) receptor] at any time point between wild-type and *Frrs1l*^−/−^ brains (Fig. 4D-F; Fig. S3C,D). To investigate further, we counted the proportion of excitatory synapses in the hippocampus and found no differences in synapse number between wild-type and *Frrs1l*^−/−^ brains (Fig. S4). Collectively, these data show that there is a significant reduction in AMPA receptor subunit levels present in adult *Frrs1l^−/−^* mice, while synaptic numbers and levels of several key synaptic markers remain unchanged. Similar changes in protein level are observed in *Frrs1l^−/−^* P0 brain, which points to a developmental defect rather than a progressive degenerative change.

### AMPA receptor glycosylation is incomplete, leading to cytoplasmic retention in *Frrs1l^−/−^* mice

Given that AMPA receptor protein levels were reduced while their transcriptional activity was unaffected, we concluded that these deficits in *Frrs1l^−/−^* brain are most likely due to specific alterations in translational or post-translational mechanisms. Interestingly, we observed that GLUA2 and GLUA4 band mobilities in western blots differ in wild-type and *Frrs1l^−/−^* mice, with a more diffuse band for GLUA2, and a slower-running band for GLUA4, in wild type compared with *Frrs1l^−/−^* (Fig. 4B,C; Fig. S3B). These differences could be related to differences in post-translational modification caused by a deficiency in this process in *Frrs1l^−/−^* mice. As part of the maturation process of the AMPA receptor complex, the glycosylation of AMPA receptors goes through a series of modifications. Initially N-linked high-mannose glycans are added to GLUA2 and GLUA4 in the ER. Subsequently, AMPA receptor complexes are transported to the Golgi apparatus, where glycans are clipped and modified to create more complex N-linked glycans. Assessing glycosylation state allows us not only to determine the extent of AMPA receptor glycosylation but also to establish the subcellular localisation of the AMPA receptor complex (Tucholski et al., 2013, 2014).

**Fig. 5.**
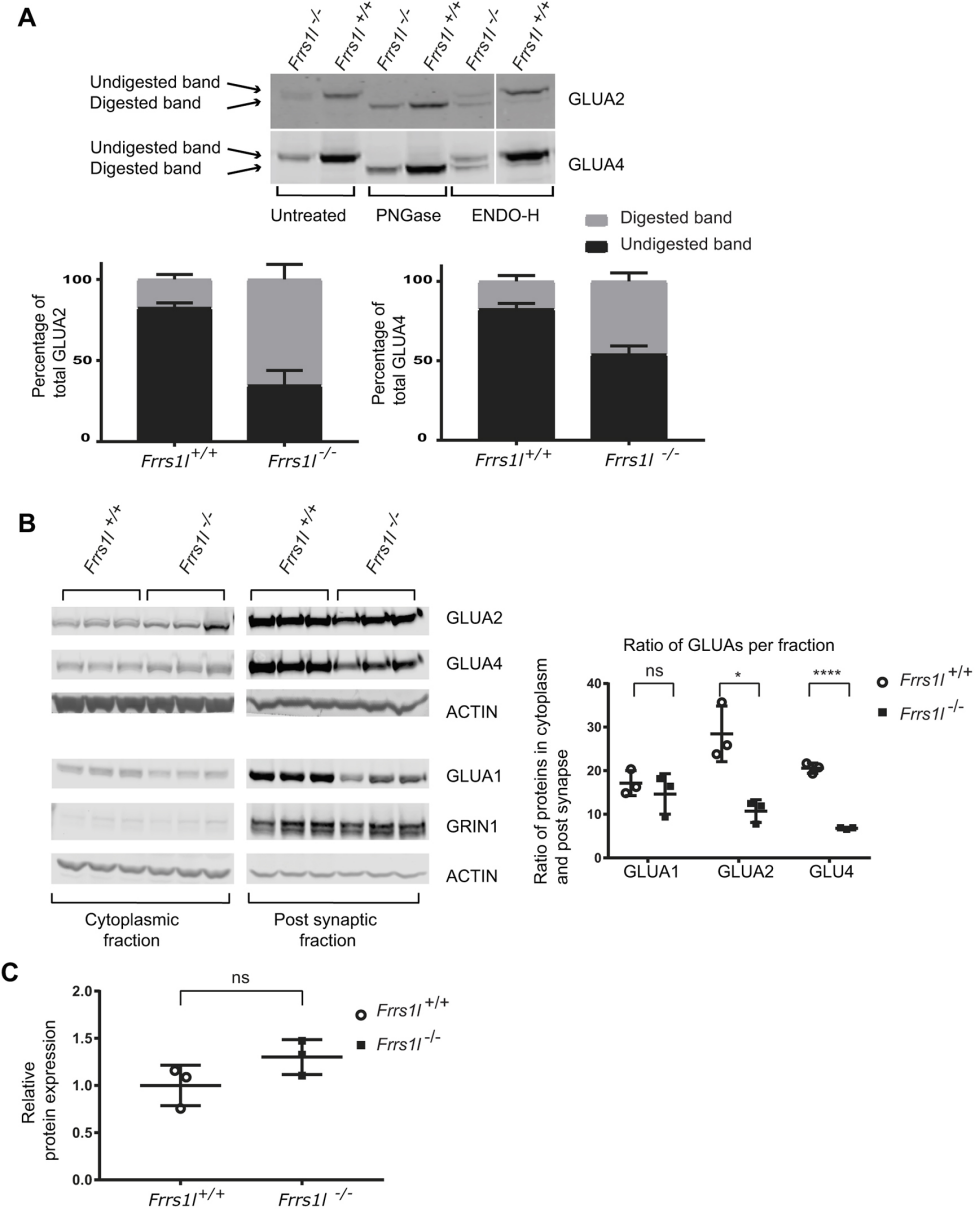
**AMPA receptors have altered glycosylation state and are mislocalised in the cytoplasm in** ***Frrs1*** **mice.** (A) Post-translational glycosylation state is altered in *Frrs1l*^−/−^, demonstrated by differential digestion with glycosylation-sensitive enzymes compared with wild-type littermates. *Frrs1l*^−/−^ show greater sensitivity of GLUA2 and GLUA4 digestion with ENDO-H (*P*<0.01); ∼18% of wild-type GLUA2 and GLUA4 is digested compared with ∼65% and 45%, respectively, in *Frrs1l*^−/−^. (B) Localisation of AMPA receptors is altered, with increased amount of AMPA receptor in the cytoplasm rather than the synapse in *Frrs1l*^−/−^. GLUA1 proportions in the cytoplasm and the synapse were not altered. (C) Overall levels of GRIN1 (NMDA receptor marker) in the post-synaptic fraction were unchanged between *Frrs1l*^−/−^ and wild-type controls. Data analysed using Student’s *t*-test (*n*=3 *Frrs1l*^−/−^, *n*=3 *Frrs1l*^+/+^). Percentage of total GLUA2 and GLUA4 data are mean±s.d. ns, non-significant; *P<0.05, ****P<0.0001.

We assessed the glycosylation status of GLUA2 and GLUA4 receptor subunits in control and mutant samples. Lysates from adult cerebellum were digested with endoglycosidase H (ENDO-H) and peptide:N-glycosidase F (PNGase) enzymes, which cleave either immature glycosylated moieties, such as high-mannose glycans, or all glycosylated forms, respectively (Fig. 5A). We found that, when digested with PNGase, all immunoreactive bands showed an apparent size shift, indicating that the subunits are typically glycosylated in both wild-type and *Frrs1l^−/−^* brain*.* When incubated with ENDO-H, in wild-type mice, only a small proportion of the GLUA2 and GLUA4 was digested, amounting to ∼18% of total protein for both. Therefore, the majority of GLUA2 and GLUA4 in wild type is insensitive to ENDO-H and thus must be maturely glycosylated. Conversely, in *Frrs1l^−/−^*, a greater proportion (*P*<0.01) of the GLUA2 and GLUA4 was digested with ENDO-H, amounting to ∼65% of GLUA2 and 45% of GLUA4 total protein. This demonstrates a higher level of immature glycosylation of the receptor subunits in the absence of FRRS1L. Based on previous work following AMPA receptor localisation and glycosylation (Tucholski et al., 2013, 2014), this result also suggests that GLUA2 and GLUA4 AMPA receptor processing is stalled at the level of the Golgi apparatus in *Frrs1l^−/−^* mice.

This increase in immaturely glycosylated AMPA receptors in *Frrs1l^−/−^* mice might result in a reduction in receptor levels at the synaptic membrane. To investigate, we carried out synaptic fractionation to determine the proportional levels of AMPA receptor in the cytoplasmic and post-synaptic membrane fractions of adult forebrain. These data show that more than double the amounts of both GLUA2 and GLUA4 are retained in the cytoplasm of *Frrs1l^−/−^* compared with wild type, leading to a reduction in the post-synaptic fraction (Fig. 5B). Interestingly, for GLUA1, levels were proportionally lower in the post-synaptic fraction in mutant brain, without evidence for retention in the cytoplasmic fraction. Additionally, NMDA receptor levels in the post-synaptic fraction were not affected by *Frrs1l* deficiency (Fig. 5C).

In conclusion, these results reveal that the loss of FRRS1L leads to a specific reduction in levels of AMPA receptor subunit proteins at the synapse *in vivo*, without obvious changes in other synaptic components. Functional FRRS1L is necessary for the mature glycosylation of at least GLUA2 and GLUA4. The mechanism through which *Frrs1l* affects glycosylation could conceivably occur through two routes, either through control of glycosylation by *Frrs1l*, or through control of earlier maturation processes, which, when disrupted, result in incomplete processing, maturation and, consequently, glycosylation. Loss of FRRS1L leads to incomplete post-translational processing of AMPA receptors, increased retention of AMPA receptors in the cytoplasmic fraction and a consequent decrease in the levels of functional AMPA receptors at the synapse.

## DISCUSSION

In previous work, we described several families with homozygous mutations in *FRRS1L* (Madeo et al., 2016). The main symptoms in the affected children are encephalopathy, epilepsy and progressive choreoathetosis. All children have severe intellectual disability with no expressive speech, impaired volitional movement and hypertonia. Initial hyperkinesia develops into chorea and finally to a hypokinetic state with seizures. This is a rare disease only recently characterised, although it is expected that there will be more cases in the near future, especially considering that the gene is now included in the screening for infantile epilepsy and dyskinesia (Genetic Testing Registry test ID GTR000551789.3).

Our data demonstrate that complete lack of *Frrs1l* has substantial effects on post-natal survival, as well as body weight, motor coordination, activity, immobility-defined sleep, effects on cognition and abnormal EEG. Anomalies are present from an early age, with no progressive deterioration, suggesting a neurodevelopmental defect, rather than an age-associated neurodegenerative disorder. Abnormalities in grip strength may be related to neurological function and correlate with hypotonia seen in human patients. The reduced grip strength might be a reflection of a neurogenic alteration rather than myogenic weakness. Similarly, human patients with mutations in FRRS1L have primarily a CNS phenotype; however, they also display hypotonia of unknown origin (Madeo et al., 2016), which corresponds with the phenotype seen in the *Frrs1l^−/−^* mice. Furthermore, there is evidence from other mouse models that neurological defects can cause reduction in grip strength through neurological mechanisms rather than muscle weakness; for example, mice with *Grin1* point mutations have NMDA receptors with reduced function and display similar reductions in grip strength to *Frrs1l^−/−^* mice (Ballard et al., 2002). Interestingly our data indicate that *Frrs1l^−/−^* mice show sleep disturbances. Although disturbances in sleep have not been documented in human patients with mutations in *FRSS1L*, it is well known that sleep disruption is often a feature of numerous neurological disorders, particularly epileptic disorders (Crespel et al., 1998; Kothare and Kaleyias, 2010). However, we do note that the sleep scoring used in this study is based upon a behaviourally defined sleep correlate for sleep rather than electrophysiological metrics. Further studies using such metrics would provide invaluable insight regarding the details of these sleep changes and how they may relate to additional phenotypes.

We report the incidental occurrence of behavioural seizures in these mice; however, we were unable to capture conclusive seizure patterns on EEG recording, which instead showed evidence of encephalographic abnormalities. Thus, all these phenotypic features resemble the symptoms seen in patients carrying homozygous mutations in the *FRRS1L* gene, making these mice a very useful model of disease. The use of these mice allowed us to provide compelling *in vivo* evidence that FRRS1L is critical for AMPA receptor complex maturation, defects in which result in dramatic phenotypic effects in mice.

In *Frrs1l^−/−^* mice, we found a highly significant reduction in the levels of core AMPA receptor proteins from birth through to adulthood, and, importantly, AMPA receptor subunits lack complex glycosylation, indicating incomplete receptor maturation. Thus, these data provide *in vivo* evidence that FRRS1L is crucial for the correct biogenesis and maturation of AMPA receptors, which elicits dramatic motor alterations, dyskinesia phenotypes and abnormal electrographical activity in *Frrs1l^−/−^* mice.

Previous work using a CRISPR/Cas9 deletion of *Frrs1l* in mouse primary neurons shows that it leads to an overall reduction in GLUA1 levels (Han et al., 2017). Here, we confirm that GLUA1 levels are lower *in vivo* but we extend this observation to other GLUAs. Crucially, we show that the low AMPA receptor levels are not a consequence of degeneration, nor are they associated with a general reduction in synaptic number in *Frrs1l^−/−^* mice. Thus, the role of FRSS1L seems to be specific in the maturation of AMPA receptor complexes. Importantly, we found that it is not only the levels of AMPA receptors that are low, but also their maturation (glycosylation) and location at synapses. AMPA receptors that are not fully glycosylated are not functional (Tomita et al., 2003; Tucholski et al., 2014), and so the presence of functional receptor complexes in the membrane is substantially deficient in mutants. We also provide evidence that FRSS1L has a critical role in the glycosylation/maturation of GLUA2 and GLUA4. It is interesting to note that the glycosylation of GLUA2 and GLUA4 is not totally abolished, indicating that there could be partial compensation for FRRS1L by another protein, or perhaps a parallel mechanism, independent of FRRS1L, which may be involved in AMPA receptor biogenesis.

Supporting these data, the *Frrs1l^−/−^* mouse phenotype resembles that of null mutations in other genes associated with the synthesis, transport and stability of AMPA receptors, such as *Cpt1c* (Carrasco et al., 2013), *Shank3* (Wang et al., 2011) and stargazin (also known as *Cacng2*) (Letts et al., 1998; Menuz and Nicoll, 2008). CPT1C is proposed to play a similar role to FRRS1L in AMPA receptor biogenesis (Brechet et al., 2017). As might be expected, many phenotypic similarities can be found in knockouts of *Cpt1c* and *Frrs1l*. Carrasco et al. (2013) demonstrated that *Cpt1c* knockout mice have poor coordination, reduced latency to fall from a rotarod, ataxia and reduced grip strength. Conversely, the *Cpt1c* knockout mice show hypoactivity, whereas we demonstrate that *Frrs1l^−/−^* mice are hyperactive. It is possible that this inconsistency is due to the different methods of activity test measurement in the two studies, or differences in background strains. However, where both studies use SHIRPA as a qualitative assessment of behaviour, there do seem to be differences in activity levels between the two strains. Furthermore, *Cpt1c* knockout mice showed a consistent reduction in two separate tests, whereas *Frrs1l^−/−^* showed a consistent increase across two tests, indicating perhaps a true difference in phenotype. Interestingly, *Cpt1c* knockout mice also show reduced levels of AMPA receptor subunit proteins, with no change in AMPA receptor gene expression by qPCR, mirroring the results seen in *Frrs1l^−/−^* mice (Fadó et al., 2015) and substantiating the argument that these two proteins are involved in the same process. Null mutations in mouse *Shank3*, a scaffold protein in the post-synaptic density, result in abnormal foot placement and reduced latency to fall from a rotarod, both of which are seen in *Frrs1l^−/−^* mice. However, *Shank3* mice also show decreased locomotion, which is contrary to the phenotype of *Frrs1l^−/−^*. The stargazin mouse has a mutation in TARP-2, an auxiliary subunit of AMPA receptors, which causes a general decrease in AMPA receptor function and phenotypes that overlap with *Frrs1l* mutants, specifically ataxia and impaired coordination (Letts et al., 1998; Menuz and Nicoll, 2008). Stargazin mice also have a similar change in glycosylation of GLUA2, as seen in *Frrs1l^−/−^*. It is noticeable that one of the main features of patients with homozygous mutations in *FRRS1L* and in the stargazin mouse model is the presence of seizures. Some incidences of behavioural seizures were observed in several *Frrs1l^−/−^* mice (one mouse was seen to have tonic clonic seizures on one occasion, another mouse had a tonic clonic seizure from which it did not recover, and other mice were seen to freeze for extended periods); such behaviours were never observed in wild-type control animals. We are aware of the limitations of the assessments conducted in this study and the need for future analysis. Further quantifiable evaluation of seizures is needed to understand the extent of the seizure phenotype in *Frrs1l^−/−^* mice.

In summary, we provide evidence for the validity of the *Frrs1l^−/−^* mouse as a model of disease, expressing phenotypic features that resemble many of the clinical symptoms in patients. At the molecular level, we have demonstrated that *FRSS1L* has a fundamental role in AMPA receptor biology, impacting the total AMPA receptor levels, as well as leading to a reduction in the proportion of AMPA receptors available at synapses. This mouse is potentially an important model to support the development of therapeutics for such patients, and is a valuable resource to further understand the complexities of AMPA receptors and glutamate signalling in the brain.

## MATERIALS AND METHODS

### Mice

All mice (*Mus musculus*) were maintained and studied in accordance with UK Home Office legislation and local ethical guidelines issued by the Medical Research Council (Responsibility in the Use of Animals for Medical Research, July 1993; Home Office licences 30/2890 and 30/3384). Mice were fed *ad libitum* on a commercial diet (SDS Rat and Mouse No. 3 Breeding diet, RM3) and had free access to water (9-13 ppm chlorine). Mice were kept under controlled light (light 07:00-19:00, dark 19:00-07:00), temperature (21±2°C) and humidity (55±10%) conditions.

*Frrs1l^tm1a/+^* mice were derived from C57BL6/NTac embryonic stem cells (Skarnes et al., 2011).The null allele (tm1b) was created by carrying out an *in vitro* fertilisation (IVF) using *Frrs1l^tm1a/+^* sperm and C57BL6/NTac oocytes. Soluble cell-permeable Cre [TAT-Cre (Tat-NLS-Cre, HTNC, HTNCre), Excellegen, Rockville, MD, USA] was added to two-cell *Frrs1l^tm1a/+^* embryos to generate the *Frrs1l^tm1b/+^*allele. The Cre excises the selection cassette and exon 3 of the *Frrs1l* gene, creating a null allele (https://www.i-dcc.org/imits/targ_rep/alleles/14093/allele-image?simple=true.jpg). Following washing to remove the soluble Cre, the IVF procedure was completed as normal. The *Frrs1l^tm1b/+^* mice were crossed to C57BL6/NTac and then intercrossed to create *Frrs1l^tm1b/tm1b^*, *Frrs1l^tm1b/+^* and *Frrs1l^+/+^* cohorts. Here, *Frrs1l^tm1b/tm1b^* is referred to as *Frrs1l^−/−^*.

### Behavioural phenotyping tests

For all behavioural phenotyping tests, mice were taken to the test room at least 20 min prior to the start of the test to acclimatise. Phenotyping equipment was cleaned with 70% ethanol/industrial methylated spirits or 2% Distel between tests. Investigators were blind to genotype during all phenotyping tests.

All phenotyping, except fear conditioning, was carried out on female mice due to issues of reduced viability. SHIRPA, grip strength and home-cage activity were assessed at 3, 6 and 9 months. All other tests were carried out on only one occasion at the ages indicated below. For behavioural tests, sample size was calculated using power equations based on previous data obtained on C57BL/6NTac mice. Sample sizes in the later time points are smaller due to the loss of several mice with welfare concerns.

#### Fear conditioning

Fear conditioning was carried out on a separate cohort of male mice prior to the rest of the study (*n*=7 C57BL/6NTac, *n*=8 *Frrs1l*^+/−^, *n*=7 *Frrs1l*^−/−^). These mice were part of the IMPC phenotyping pipeline; the remainder of the IMPC phenotyping data are published on the IMPC data portal (http://www.mousephenotype.org; Koscielny et al., 2014).

#### Open-field activity

Open-field activity was used to assess locomotion in a novel environment. At 10 weeks of age (±1 week), mice were placed in square arenas (44×44 cm) in a small testing room. A minimum of two and a maximum of four mice were tested at one time, one mouse per arena. Lighting was set at 150-200 lux. Mice were video tracked for 20 min and data analysed using Ethovision XT software (Noldus, Wageningen, The Netherlands) and parameters such as distance moved, velocity and duration moving were recorded in various zones over the entire 20-min period (Joyce et al., 2016) (*n*=7 *Frrs1l*^+/+^, *n*=10 *Frrs1l*^−/−^).

#### SHIRPA

A semi-quantitative assessment was carried out using a modified SHIRPA protocol. Behaviour and dysmorphology parameters were recorded as previously described (Masuya et al., 2005) (*n*=8 *Frrs1l*^+/+^, *n*=9 *Frrs1l*^−/−^ at 3 months; *n*=8 *Frrs1l*^+/+^, *n*=9 *Frrs1l*^−/−^ at 6 months; *n*=8 *Frrs1l*^+/+^, *n*=7 *Frrs1l*^−/−^at 9 months).

#### Grip strength

Grip strength was assessed at 13 weeks of age (±1 week) using the Grip Strength Test (BioSeb, Chaville, France). Readings were taken from all four paws, three times per mouse at each age, as per the manufacturer's instructions (Joyce et al., 2016) (*n*=8 *Frrs1l*^+/+^, *n*=8 *Frrs1l*^−/−^ at three months; *n*=8 *Frrs1l*^+/+^, *n*=8 *Frrs1l*^−/−^ at 6 months; *n*=6 *Frrs1l*^−/−^, *n*=6 *Frrs1l*^+/+^ at 9 months).

#### Home-cage analysis

Group-housed animals were monitored as described (Bains et al., 2016). Briefly, group-housed mice were tagged with RFID microchips at 9 weeks of age and placed in a Home Cage Analysis system (Actual Analytics, Edinburgh, UK), which captured mouse behaviour using both video tracking and location tracking using RFID coordinates (Bains et al., 2016) (*n*=8 *Frrs1l*^+/+^, *n*=8 *Frrs1l*^−/−^ at all time points).

#### Locotronic

Paw placement was analysed using Locotronic (Intelli-Bio, Seichamps, France) at 13 weeks of age (±1 week). Briefly, animals were assessed as they move along a corridor with a horizontal ladder as its base. Animals were motivated to travel from a lighter starting area at one end to a darker finish area at the other end (bars, 3 mm diameter; spaced by 7 mm). Infrared sensors above and below each bar space recorded any errors of paw placement. Trials were discounted if the mouse took more than 30 s to move to the finish after exiting the start area (*n*=8 *Frrs1l*^+/+^, *n*=7 *Frrs1l*^−/−^).

#### Rotarod

To assess coordination and motor learning, 22-week-old mice (±2 weeks) were placed on an accelerating rotarod (Ugo Basile, Gemonio, Italy), with rotor speed increasing from 4 rpm up to 40 rpm over a 5-min period. The time taken for the mouse to fall from the rod was recorded. This was repeated three times in 1 day, with a 15-min inter-trial interval (Corrochano et al., 2012) (*n*=8 *Frrs1l*^+/+^, *n*=9 *Frrs1l*^−/−^).

#### Y-maze

A forced alternation Y-maze test was used to evaluate short-term working memory in mice at 14-20 weeks of age. Mice were placed in a Y-maze with access to one arm blocked; they were then free to explore the start arm and the ‘familiar’ arm for 10 min. Mice were returned to the home cage for a 2-min inter-trial interval, during which the maze was cleaned to remove odour cues. The mice were then returned to the maze, with access to all three arms open for 5 min. Mice were video tracked at all times using Ethovision software (Noldus) (Sanderson and Bannerman, 2012) (*n*=7 *Frrs1l*^+/+^, *n*=7 *Frrs1l*^−/−^).

#### Motor function assessment by wheel running

For further assessment of motor function, 45- to 50-week-old mice were singly housed and placed in cages containing a running wheel as previously described (Mandillo et al., 2014) (TSE Systems, Bad Homburg, Germany). Number of rotations, time running, number of bouts and speed were measured. After 2 weeks with the standard wheel, this was replaced with a complex wheel that had specific rungs removed in order to test coordination and learning. Parameters were recorded for a further week (*n*=5 *Frrs1l*^+/+^, *n*=5 *Frrs1l*^−/−^).

#### Passive infrared screen for immobility-defined sleep (PIR)

At 1 year, mice were analysed for circadian activity and immobility defined sleep using the COMPASS system as described (Brown et al., 2016). Mice were individually housed and data captured for 5 days in a 12 h:12 h light/dark cycle, followed by 9 days in constant darkness. Data analysis was performed using custom python scripts and Excel sheets, developed in house. Circadian analysis was performed by converting activity data from PIR to AWD files for analysis on Clocklab (Actimetrics, Wilmette, IL, USA) or Actiwatch Sleep analysis software (CamNtech, Cambridge, UK) (*n*=5 *Frrs1l*^+/+^, *n*=6 *Frrs1l*^−/−^).

#### qPCR analysis

RNA extraction from P0 brain tissue or cerebellum of 14-month-old mice was performed using an RNeasy kit (Qiagen) (*n*=5 *Frrs1l*^+/+^, *n*=5 *Frrs1l*^−/−^ at P0; *n*=4 *Frrs1l*^+/+^, *n*=4 *Frrs1l*^−/−^ at 14 months). Complementary DNA (cDNA) synthesis was performed using a High-Capacity cDNA RT kit (Thermo Fisher Scientific) starting with 2 µg total RNA. cDNA for qPCR amplification was used at a final concentration of 10 ng per well. All the reactions were run in triplicate. Fast Sybr Green Mastermix from Thermo Fisher Scientific was used and the reactions had a final volume of 20 µl. Primers were at a final concentration of 360 nM. Primers were designed to span exon-exon boundaries and are listed in Table S1. Fold changes were calculated using the 2-ddCt method using 7500 Software v2.0.6 (Thermo Fisher Scientific) and normalised using S16 endogenous reference genes relative to wild-type genotype (Livak and Schmittgen, 2001).

#### Immunoblot analysis

Adult cerebellum or P0 whole brains were bisected and one fraction homogenised in RIPA buffer [150 mM NaCl, 1% NP40, 0.5% Na deoxycholate, 0.1% sodium dodecyl sulfate (SDS), 50 mM Tris-HCl, pH 7.5] with phosphatase and protease inhibitor cocktails (Roche), using lysing matrix tubes D (MP Biomedicals, Eschwege, Germany) and a Fast-Prep-24 homogeniser at 4°C. Homogenates were centrifuged at 12,000 ***g*** at 4°C for 20 min. Then, 30 μg of soluble fractions were resolved by SDS–PAGE (NUPAGE system, Invitrogen) and transferred to nitrocellulose membranes (Millipore) for western blot analysis. The following primary antibodies were used: rabbit monoclonal anti-b-actin (1/3000, Sigma-Aldrich, A2066), mouse anti-α tubulin (1/3000, Sigma-Aldrich, T9026), rabbit anti-GLUA1 (1/1000, Millipore, 1504), mouse anti-GLUA2 (1/800, Millipore, MAB397), rabbit anti-GLUA4 (1/1000, Millipore, AB1508), mouse anti-CAMKII (1/200, Proteintech, 20665-1), mouse anti-SNAP25 (1/500, BioLegend, 836304), rabbit anti-PSD95 (1/1000, Cell Signaling Technology, 3450T) and mouse anti-GRIN1 (for NMDA receptor, 1/1000, Novus, NB300-118).

Protein was visualised using anti-mouse (P/N 926-68070) or anti-rabbit (P/N 926-32211) secondary antibodies IRDye® (Li-Cor Biosciences) at 1:10,000 dilutions and quantified using the scanning infrared Odyssey imaging system CX (Li-Cor Biosciences).

All antibodies used have been previously validated and published, and were purchased from commercial suppliers.

#### Glycosylation assay

Bisected adult cerebellum was homogenised in RIPA buffer as above, and 60 µg of the soluble fraction was denatured with glycoprotein denaturing buffer for 10 min at 100°C, followed by immediate immersion in ice. Samples were divided into three aliquots and glycobuffer added, followed by ENDO-H (QABio, E-EH02), PNGase (New England Biolabs, P0704S) or water. All samples were incubated at 37°C for 1 h. Half of each sample, containing 10 μg protein, was resolved by SDS–PAGE (NUPAGE system, Invitrogen) and transferred to nitrocellulose membranes for western blot analysis.

#### Post-synaptic fractionation enrichment

Synaptic fractionation was conducted following a modified protocol using a single cerebral hemisphere, homogenised using a Dounce homogeniser in Syn-PER™ Synaptic Protein Extraction Reagent (Thermo Fisher Scientific) with phosphatase and protease inhibitor cocktails (Roche). Homogenates were cleared by centrifugation at 1200 ***g*** for 10 min and then at 15,000 ***g*** for 20 min at 4°C. The supernatant contains the cytosolic fraction and the pellet the crude synaptosomal fraction. The pellet was resuspended in syn-PER lysis buffer with 0.1 mM CaCl2 and 2% Triton X-100, 40 mM Tris-HCl, pH 6, and incubated on ice, with gentle agitation, for 30 min. Following a centrifugation at 40,000 ***g*** for 30 min at 4°C, the pellet was washed with 1% Triton X-100, 20 mM Tris-HCl, pH 6. Then, the sample was centrifuged again, resuspended in 1% Triton X-100, 20 mM Tris-HCl, pH 8, and incubated on ice with gentle agitation for 30 min. After another centrifugation at 40,000 ***g*** for 30 min at 4°C the pellet, containing the post-synaptic density, was resuspended in 1% Triton X-100, 20 mM Tris-HCl, pH 8, precipitated by adding ten volumes of ice-cold acetone at −20°C overnight, and subsequently centrifuged at 15,000 ***g*** for 30 min, at 4°C. The pellet containing the post-synaptic fraction was resuspended in 5% SDS. After protein quantification with a DC assay (Bio-Rad), 15 μg of protein from the post-synaptic fraction and 20 μg from the cytoplasmic fraction were resolved in pre-cast 3-8% SDS–PAGE gels and transferred to nitrocellulose membrane (Invitrogen) for western blot analysis.

#### Synapse counts

Formalin-fixed wax-embedded sections from adult whole brain were dewaxed, and the antigen was unmasked using sodium citrate buffer solution (pH 6.0) at 80°C for 30 min. Sections were then washed in phosphate-buffered saline (PBS, pH 7.4) and processed for immunofluorescence. After a blocking step in PBS containing 0.05% Triton X-100 and 10% normal goat serum, sections were incubated overnight at 4°C with the antibodies anti-VGLUT1 (1:200, Synaptic System). Antibody was diluted in PBS with 3% normal goat serum and 0.05% Triton X-100. Sections were then washed in PBS (4×10 min) and incubated for 1 h at room temperature with a secondary anti-rabbit antibody conjugated to Alexa Fluor 488 (Invitrogen). After several PBS rinses, sections were mounted on glass slides and observed with a Zeiss LSM 700 confocal microscope (Carl Zeiss AG). Confocal *z*-stacks covering the whole depth of the slices (1024×1024 pixels) spaced by 1.05 µm were acquired at 63×. VGLUT1-positive puncta were analysed on confocal images using Fiji software (Schindelin et al., 2012). Caudal sections were used to analyse both the stratum oriens and radiatum of the CA1 region of the hippocampus.

#### Telemetry EEG

EEG measurements were conducted in male mice, ∼30 g in weight, and aged between 4 and 6 months (*n*=3 *Frrs1l^−/−^*, *n*=2 *Frrs1l^/+/+^*, *n*=2 C57BL/6NTac). Wireless EEG transmitters (A3028A, Open Source Instruments, Watertown, MA, USA) were implanted subcutaneously with a subdural intracranial recording electrode positioned above the right frontal lobe (0.5 mm anterior to Bregma and 0.5 mm medial lateral). The second electrode was implanted between the frontal and occipital lobes, above the right motor cortex (1-1.5 mm posterior to Bregma and 3-4 mm medial lateral). The animals were able to freely move while assessed in their home-cage environment for 5-21 days. EEG was recorded after three or more post-surgery recovery days to ensure no residual anaesthesia effect. Dataquest software (Neuroachieve v. 8.5.20, Open Source Instruments) was used to acquire and analyse the EEG data. EEG activity was sampled at 512 Hz, then filtered between 0.3 Hz and 160 Hz. Representative high-amplitude signals were screened visually, and fast Fourier transform was used to determine EEG amplitude and frequency.

#### Statistical analysis

Estimates of Mendelian inheritance of all genotypes were assessed using a chi-squared test. Analysis of categorical data from SHIRPA was completed using Fisher’s exact test. Grip strength test, rotarod, home-cage analysis, time spent asleep, sleep bouts over time, sleep fragmentation and motor function wheel-running data were assessed using repeated measures analysis of variance (ANOVA) with Sidak post hoc analysis. Body weight was assessed using two-way ANOVA with post hoc comparisons. Forced alternation Y-maze data were assessed using the Student's *t*-test. Open-field data were analysed using the Student's *t*-test with Welch's correction for unequal variance. GraphPad Prism and R software (www.r-project.org) were used for statistical analysis.

All graphs show absolute values with mean and s.d., unless specified.

## Supplementary Material

Supplementary information
