# Supplementary material for: Loss of Frrs1l disrupts synaptic AMPA receptor function, and results in neurodevelopmental, motor, cognitive and electrographical abnormalities
Source: Dis Model Mech. 2019 Feb 22;12(2):dmm036806. doi: 10.1242/dmm.036806 (PMC6398485; doi:10.1242/dmm.036806)
Supplement: Supplementary information [file dmm-12-036806-s1.pdf]

Table S1. ***Primer sequences for gene expression analysis by qPCR.***

Gene		Primer sequence
Gria 1	Fwd	TCCCCAACAATATCCAGATAGGG
	Rev	AAGCCGCATGTTCCTGTGATT
Gria 2	Fwd	TTCTCCTGTTTTATGGGGACTGA
	Rev	CTACCCGAAATGCACTGTATTCT
Gria 3	Fwd	ACCATCAGCATAGGTGGACTT
	Rev	ACGTGGTAGTTCAAATGGAAGG
Gria 4	Fwd	GTTTTCTGGATTTTGGGGACTCG
	Rev	AAGAGACCACCTATTTGAACGC
Frrs1l Exon 1-2	Fwd	CCAGCGAGTTCTACGACCTG
	Rev	TCCCGTATCTGAAGCATCCC
Frrs1l Exon 2-3	Fwd	GTGGACCCATTCGCCAAAAT
	Rev	CGCCTATCATCCGGTAGCTA
Frrs1l Exon 4-5	Fwd	TGTTCCCAGGGATGAAACGA
	Rev	AAGCCGGTGGAGAGTCTATG

Disease Models & Mechanisms • Supplementary information

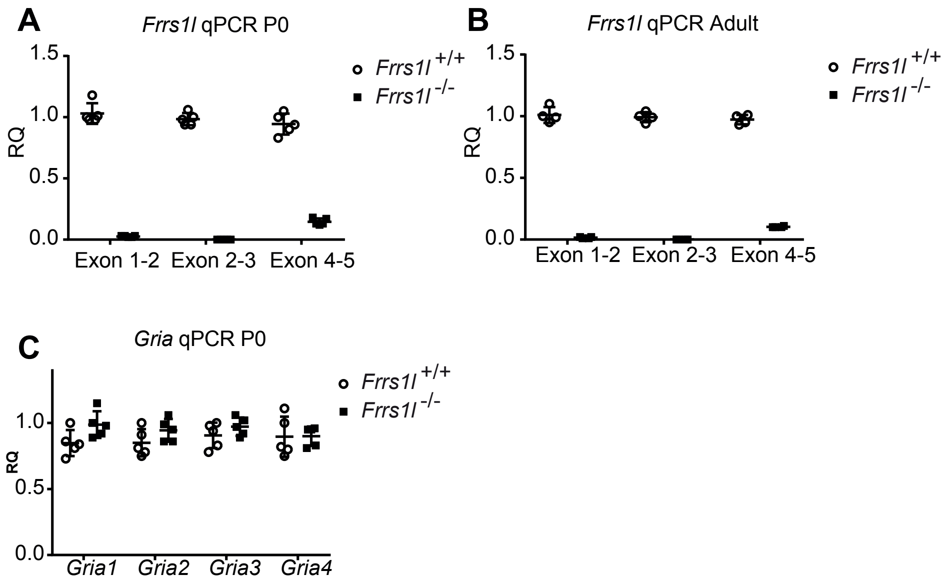

Fig. S1. *Frrsl1*$^{-/-}$ mice have no expression of Frrs1l in both P0 pups and adults brain (A and B). No changes in Gria expression are seen at P0 in *Frrsl1*$^{-/-}$ mice compared to wild-type controls.

Disease Models & Mechanisms • Supplementary information

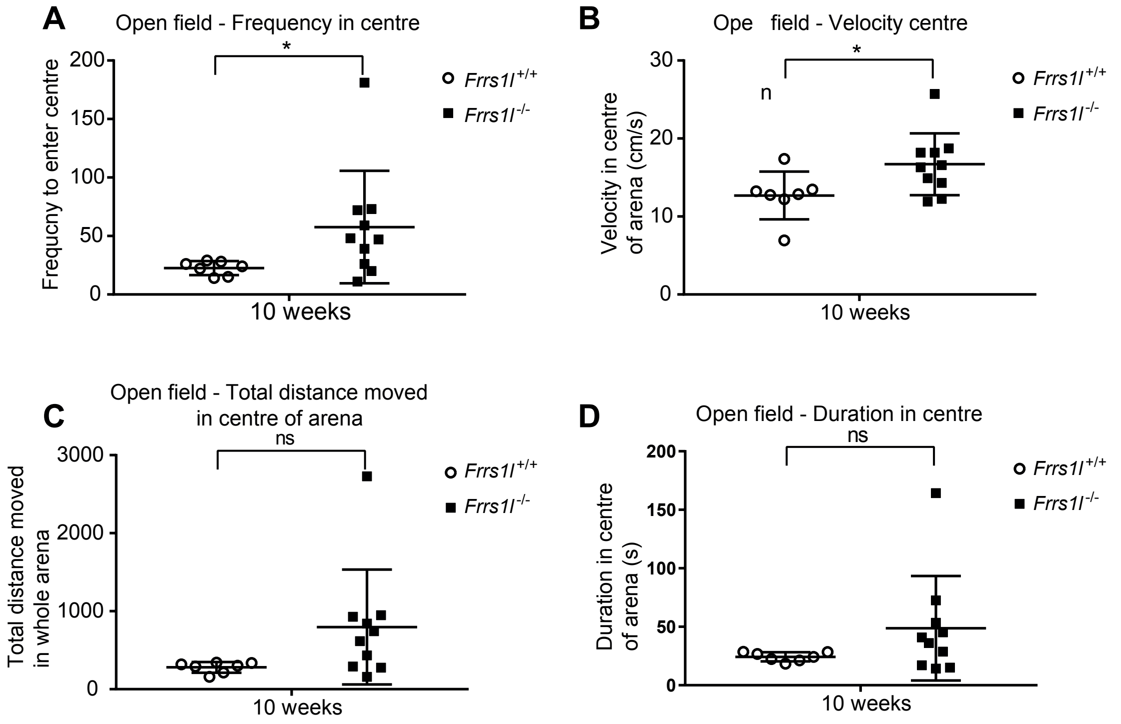

Fig. S2. *Frrsl1*$^{-/-}$ mice show changes in some but not all anxiety related parameters in open field.

Disease Models & Mechanisms • Supplementary information

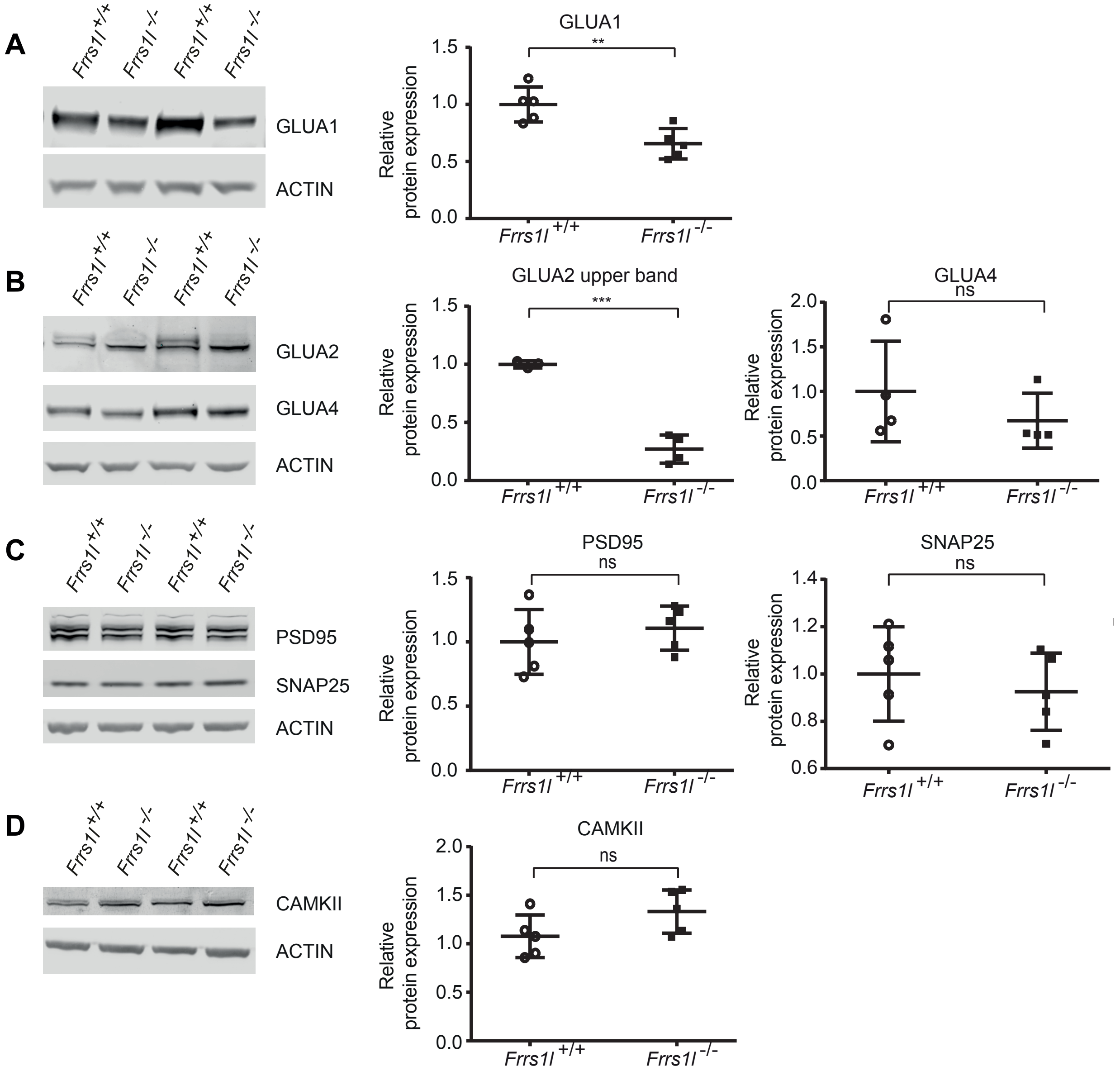

Fig. S3. *Frrs1l$^{-/-}$ P0 brains show alterations in levels of AMPA receptor proteins.* *Frrs1l$^{-/-}$ P0 brains have significantly less GLUA1 (p<0.01), whilst GLUA2 and GLUA4 total levels are unchanged between Frrs1l-/- and wild-types. However two distinct bands are present in wild-type GLUA2 with the upper band significantly reduced in Frrs1l$^{-/-}$ (p<0.001). Levels of other synaptic proteins PSD95, SNAP25 and CAMKII are the same in both Frrs1l$^{-/-}$ and wild types. Data analysed using t-test (n=5 Frrs1l-/-, n=5 Frrs1l+/+) for all except GLUA2 (n=3 Frrs1l$^{-/-}$, n=4 Frrs1l$^{+/+}$) and GLUA4 (n=4 Frrs1l$^{-/-}$, n=4 Frrs1l$^{+/+}$).*

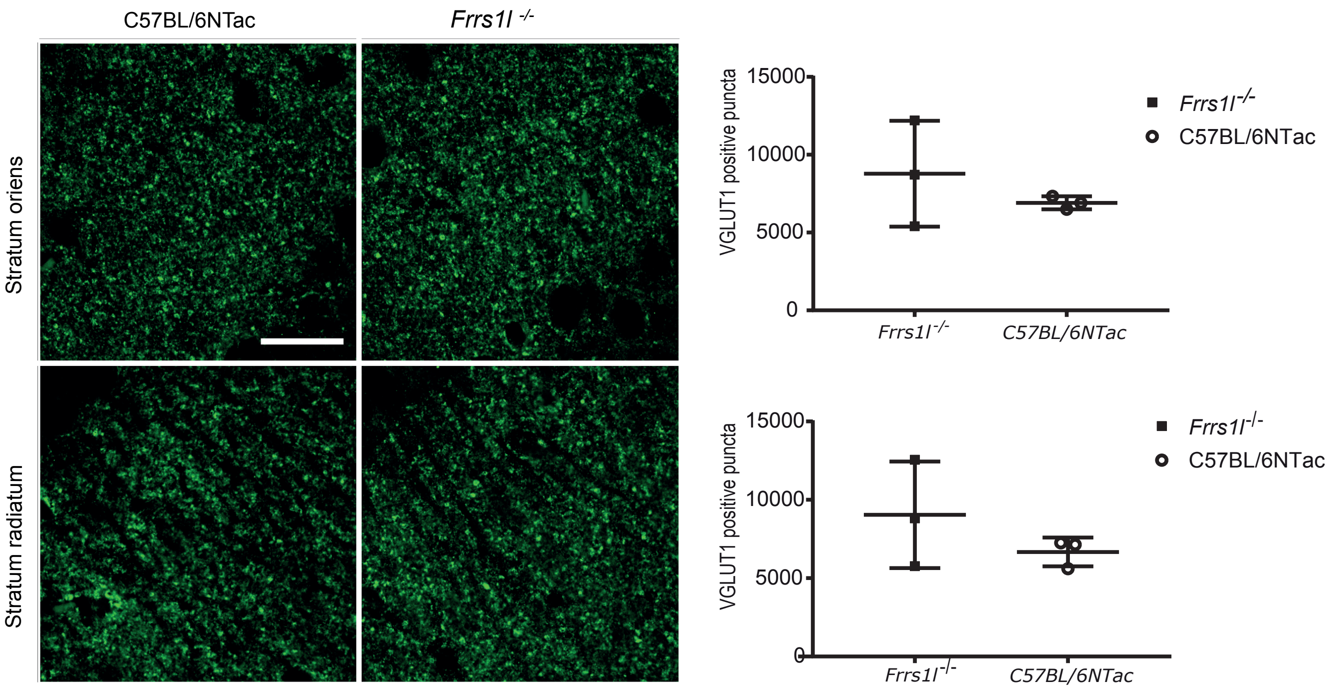

Fig. S4. *No differences were found in excitatory synapse number in the hippocampus of Frrs1l$^{-/-}$ mice compared to C57BL/6NTac controls.*
